# Associations of lifestyle activities and a heathy diet with frailty in old age: a community-based study in Singapore

**DOI:** 10.18632/aging.102615

**Published:** 2020-01-02

**Authors:** Xiu Wang, Yanxia Lu, Chunbo Li, Anis Larbi, Liang Feng, Qingfeng Shen, Mei Sian Chong, Wee Shiong Lim, Lei Feng

**Affiliations:** 1 Department of Neurology, Beijing Chuiyangliu Hospital, Beijing, PR China; 2 Department of Clinical Psychology and Psychiatry/School of Public Health, Zhejiang University College of Medicine, Hangzhou, China; 3 Biology of Ageing Laboratory, Singapore Immunology Network (SIgN), Agency for Science Technology and Research (A*STAR), Singapore; 4 Shanghai Key Laboratory of Psychotic Disorders, Shanghai Mental Health Center, Shanghai Jiao Tong University School of Medicine, Shanghai, PR China; 5 Institute of Psychology and Behavioral Science, Shanghai Jiao Tong University, Shanghai, PR China; 6 Program in Health Services and Systems Research, Duke-NUS Medical School, Singapore; 7 Department of Geriatric Psychiatry, Xuzhou Oriental people’s Hospital, China; 8 Geriatric Education and Research Institute, Singapore; 9 Department of Geriatric Medicine, Institute of Geriatrics and Active Ageing, Tan Tock Seng Hospital, Singapore; 10 Department of Psychological Medicine, National University of Singapore, Singapore; 11 Centre for Healthy Ageing, National University Health System, Singapore

**Keywords:** frailty, lifestyle activity, healthy diet, risk factors, gender differences

## Abstract

Frailty is an age-related state characterized by a reduced physiological reserve, and is associated with adverse health outcomes in the elderly. We analyzed the data from 895 adults aged 60 years and above, and investigated the relationships between midlife and late-life social activities, intellectual activities, working hours, and dietary habits and frailty status. Participation in social or intellectual activities in late life was less prevalent among those who were frail than among those who were robust. A greater proportion of those who were frail had worked long hours in midlife. After adjustment for confounders, participating in social activities or intellectual activities in late life was associated with a reduced risk for prefrailty and frailty, while working long hours in midlife was associated with a higher risk for frailty. The risk of frailty decreased with increasing healthy diet scores in midlife and late life. When the results were stratified by gender, late-life participation in social activities and midlife or late-life participation in intellectual activities correlated negatively with prefrailty/frailty only in women. Our study suggests that social and intellectual activities are inversely associated with frailty status, but the association seems to differ based on gender.

## INTRODUCTION

Population aging has posed great public health challenges worldwide [1]. Although chronological age correlates with biological age, there can be significant differences in health and functional status among individuals with the same chronological age. Frailty is a concerning state in which the physiological reserve is reduced, making individuals more vulnerable to stressors and adverse health outcomes such as falls, comorbidities, disabilities and mortality [2, 3]. Frailty is a dynamic process that can worsen or be reversed over time [4–8]. The identification of modifiable risk factors and protective factors for frailty is essential for healthcare planning and targeted intervention development. However, the factors associated with frailty are not well understood.

Numerous studies have indicated that active participation in leisure activities [9, 10], social activities [11–16] or intellectual activities [17–20] can improve the cognitive reserve [9–11, 15, 17, 18], enhance mental health [13, 16], reduce functional disabilities [14, 20] and delay mortality [11, 12, 19]. On the other hand, working long hours (i.e., more than 40 hours per week or eight hours per day) appears to be associated with deleterious effects such as depression, anxiety, sleep disorders and coronary heart disease [21]. Despite the evidence that engagement in various lifestyle activities influences health outcomes, few studies have examined frailty as an outcome variable. Additionally, although the diet is a frequently studied lifestyle factor, its impact on frailty has not received much attention [22, 23].

Understanding the associations of health-related lifestyle factors with frailty could help healthcare providers formulate screening and delivery strategies for health and social care. In this study, we evaluated the associations of frailty status with midlife and late-life social activities, intellectual activities, working hours and dietary consumption patterns in a community-based sample of older adults in Singapore.

## RESULTS

The final sample included in this analysis comprised 895 participants aged 60 years and older, among whom 95.8% were ethnic Chinese. The mean age of the participants was 67.9 years (range 60–93 years), with a standard deviation of 5.8 years. Among the participants, 70.8% were females, and only 28.6% had completed secondary school or higher education.

### Demographics, medical comorbidities, healthy diet scores and frailty

The demographic characteristics associated with frailty status are shown in Table 1. Among all the participants, the prevalence of frailty was 5.0% (n=45), while the prevalence of prefrailty was 51.1% (n=457). The participants with frailty were older, had a lower education level, and were more likely to live in lower-end housing, live alone and be single or widowed (*p*<0.05 for each comparison). There was no statistically significant difference in the prevalence of frailty between women and men (*p*=0.576).

**Table 1:** Sociodemographic information, medical conditions and healthy diet score of participants with different frailty status.

	**Robust (N=393)**	**Pre-frail (N=457)**	**Frail (N=45)**	p*
Age, years	66.6±5.0	68.6±5.9	71.7±7.7	<0.001**
Age group				
60-69	288(73.3)	283(61.9)	21(46.7)	<0.001
70-79	98(24.9)	149(32.6)	17(37.8)	
≥80	7(1.8)	25(5.5)	7(15.6)	
Sex				
Male	116(29.5)	135(29.5)	10(22.2)	0.576
Female	277(70.5)	322(70.5)	35(77.8)	
Education levels				
No education	119(30.3)	180(39.4)	24(53.3)	0.001
Primary	137(34.8)	166(36.3)	13(28.9)	
Secondary/higher	137(34.8)	111(24.3)	8(17.8)	
Housing				
1-3 room	53(13.5)	85(18.7)	12(26.7)	0.001
4-5 room	283(72.2)	340(74.7)	30(66.7)	
High-end housing	56(14.3)	30(6.6)	3(6.7)	
Marital status				
Married	281(71.7)	306(67.4)	22(48.9)	0.006
Single/widowed	111(28.3)	148(32.6)	23(51.1)	
Living condition				
Alone	23(5.9)	37(8.1)	6(13.3)	0.017
With spouse	274(70.1)	289(63.5)	21(46.7)	
With children or other	94(24.0)	129(28.4)	18(40.0)	
Occupation				
Retired	215(55.0)	225(49.4)	26(60.5)	0.089
Current employment	68(17.4)	90(19.8)	2(4.6)	
Housewife	108(27.6)	140(30.8)	15(34.9)	
Number of Comorbidity			
0-1	152(38.7)	168(36.8)	10(22.2)	0.004
2-3	181(46.0)	194(42.4)	18(40.0)	
>3	60(15.3)	95(20.8)	17(37.8)	
Hypertension	194(49.4)	232(50.8)	23(51.1)	0.913
High cholesterol	214(54.5)	238(52.1)	26(57.8)	0.656
Diabetes	60(15.3)	88(19.3)	10(22.2)	0.224
Stroke	7(1.8)	24(5.3)	3(6.7)	0.018
Cardiac disease	26(6.6)	48(10.5)	3(6.7)	0.117
Cataracts/glaucoma	115(29.3)	171(37.4)	28(62.2)	<0.001
Kidney failure	0	6(1.3)	1(2.2)	0.023***
Asthma	21(5.3)	11(2.4)	4(8.9)	0.022
COPD	1(0.3)	2(0.4)	0(0)	-
Arthritis	56(14.2)	66(14.4)	11(24.4)	0.181
Osteoporosis	25(6.4)	25(5.5)	4(8.9)	0.613
Hip fracture	1(0.3)	4(0.9)	1(2.2)	0.146***
GIP	37(9.4)	41(9.0)	5(11.1)	0.890
Thyroid problems	35(8.9)	29(6.3)	3(6.7)	0.373
Cancer	17(4.3)	28(6.1)	4(8.9)	0.301
GAI≥10	0	17(3.7)	1(2.2)	0.001
GAI score	0.7±1.65	1.5±2.90	1.8±2.79	<0.001**
SM-MMSE≤23	9(2.3)	38(8.3)	7(15.6)	<0.001
SM-MMSE score	28.2±1.94	27.6 ±2.66	27.0±3.77	0.040**
HDS in midlife	15.0±3.0	14.6±2.7	13.7±2.6	0.001**
HDS in late-life	15.3±2.5	14.7±2.8	14.0±3.1	<0.001**

Shown are numbers (%), mean±SD unless stated otherwise.

*P-value obtained using Chi-square test.

** P-value obtained using Kruskal Wallis Test.

*** P-value obtained using Fisher’s exact tests.

SD, standard deviation; GAI, Geriatric Anxiety Inventory; SM-MMSE, Singapore Modified Mini-Mental State Examination; COPD, Chronic Obstructive Pulmonary disease; GIP, Gastrointestinal problems; HDS, healthy diet score.

Table 1 also displays the frequency distributions of chronic diseases, cognitive impairment and anxiety in participants with different frailty statuses. Of the frail participants, 77.8% had two or more chronic diseases. Frailty was not associated with the presence of hypertension, high cholesterol, diabetes, cardiac disease, arthritis, osteoporosis, hip fracture, gastrointestinal problems, thyroid problems or cancer. On the other hand, frailty was significantly associated with the presence of stroke (*p*<0.05), cataracts/glaucoma (*p*<0.001), kidney failure (*p*<0.05) and asthma (*p*<0.05). Cognitive impairment (a score ≤ 23 on the Singapore Modified version of the Mini Mental State Examination [SM-MMSE]) was significantly more prevalent among frail participants (15.6%) than among their robust counterparts (2.3%). As for anxiety, 18 individuals had Geriatric Anxiety Inventory scores ≥ 10, and all of these subjects were in the prefrail and frail groups. The healthy diet score (HDS) was lower in the prefrail and frail groups than in the robust group at midlife (*p*=0.001) and late life (*p*<0.001). These data suggest that frailty is associated with a high total number of medical comorbidities and with the presence of individual medical comorbidities, cognitive impairment and poor mental health.

### Lifestyle activities and frailty

The associations of midlife and late-life activities with frailty are shown in Table 2. Greater proportions of prefrail and frail subjects than robust subjects were isolated from social and intellectual activities. Specifically, 48.8% of the frail and 38.8% of the prefrail subjects did not participate in midlife social activities, compared with 32% of the robust subjects (*p*=0.025). Furthermore, 35.6% of the frail and 24.8% of the prefrail subjects did not participate in late-life social activities, compared with 13.7% of the robust subjects (*p*<0.001). Regarding intellectual activities, 47.7% of the frail and 44.4% of the prefrail subjects were midlife nonparticipants, compared with 32.9% of the robust subjects (*p*=0.002). Likewise, 54.5% of the frail and 39.2% of the prefrail subjects were late-life nonparticipants in intellectual activities, compared with 26.3% of the robust subjects (*p*<0.001). A greater percentage of frail participants than robust participants had worked long hours in midlife (80% vs. 59.1%, *p*=0.024), while this effect was not observed in those who were working more than nine hours per day in late life (*p*=0.828). Smoking and alcohol intake were not associated with frailty.

**Table 2:** The comparisons of participants with various lifestyle activity by frailty status.

	**Robust (%)**	**Prefrail (%)**	**Frail (%)**	p*
Social activities in midlife			
nonparticipation	125 (32.0)	177 (38.8)	21 (48.8)	0.025
participation	266 (68.0)	279 (61.2)	22 (51.2)	
Social activities in late-life			
nonparticipation	54 (13.7)	113 (24.8)	16 (35.6)	<0.001
participation	339 (86.3)	343 (75.2)	29 (64.4)	
Intellectual activities in midlife		
nonparticipation	127 (32.9)	202 (44.4)	21 (47.7)	0.002
participation	259 (67.1)	253 (55.6)	23 (52.3)	
Intellectual activities in late-life		
nonparticipation	103 (26.3)	178 (39.2)	24 (54.5)	<0.001
participation	289 (73.7)	276 (60.8)	20 (45.5)	
Work more than 9 hours in midlife			
nonparticipation	159 (40.9)	182 (39.9)	9 (20.0)	0.024
participation	230 (59.1)	274 (60.1)	36 (80.0)	
Work more than 9 hours in late-life			
nonparticipation	364 (92.9)	418 (91.9)	41 (91.1)	0.828
participation	28 (7.1)	37 (8.1)	4 (8.9)	
Smoker in midlife				
smoker	44 (11.3)	63 (13.8)	8 (17.8)	0.336
non-smoker	345 (88.7)	392 (86.2)	37 (82.2)	
Smoker in late-life				
smoker	18 (4.6)	30 (6.6)	4 (8.9)	0.312
non-smoker	374 (95.4)	426 (93.4)	41 (91.1)	
Drink alcohol in midlife				
alcohol consumer	58 (14.9)	71 (15.6)	6 (13.3)	0.901
non-alcohol consumer	330 (85.1)	383 (84.4)	39 (86.7)	
Drink alcohol in late-life				
alcohol consumer	38 (9.7)	44 (9.7)	3 (6.7)	0.800
non-alcohol consumer	355 (90.3)	411 (90.3)	42 (93.3)	

Both ‘smoking’ and ‘drink alcohol’ were defined as more than once a month.

The frequencies of participation were recorded on a 5-point scale: 1. never or rarely, 2. more than once a month but less than once a week, 3. one to three times a week, 4. four to six times a week, 5. daily. Nonparticipation was defined as level 1. Participation was defined from level 2 to level 5.

*P-value was obtained using Chi-square test.

We then performed multinomial logistic regression analyses to quantify the relationships of lifestyle activities and the HDS in midlife and late-life with the presence of frailty (Table 3**)**. Late-life social and intellectual activities were found to protect against prefrailty and frailty. For those who participated in late-life social activities compared with those who did not, the adjusted odds ratio (OR, controlled for relevant covariates) for prefrailty was 0.43 (95% confidence interval [CI] 0.29-0.63) and the adjusted OR for frailty was 0.21 (95% CI 0.10-0.45). For those who participated in late-life intellectual activities compared with those who did not, the adjusted OR for prefrailty was 0.57 (95% CI 0.42-0.77) and the adjusted OR for frailty was 0.35 (95% CI 0.18-0.69). Midlife intellectual activity only correlated negatively with prefrailty (adjusted OR=0.60, 95% CI 0.45-0.81, *p*=0.001). Working long hours in midlife was associated with an increased risk of frailty (adjusted OR=2.96, 95% CI 1.34-6.57, *p*=0.007), but not prefrailty (adjusted OR=1.02, 95% CI 0.76-1.37, *p*=0.875). Furthermore, the probability of frailty decreased by approximately 15% (OR=0.86, 95% CI 0.77-0.96, *p*=0.012; OR=0.85, 95% CI 0.75-0.95, *p*=0.008) with each one-point increase in the HDS in midlife and late-life.

**Table 3:** Association of lifestyle activities and healthy diet score with prefrailty and frailty.

**Lifestyle activities, yes vs no**	**Prefrailty**					**Frailty**				
	**N**	**OR (95%CI)**	***p***	Adjusted OR* (95%CI)	***p***	**N**	**OR (95%CI)**	***p***	Adjusted OR* (95%CI)	***p***
Social activities in midlife	279	0.74 (0.56-0.98)	0.038	0.76 (0.56-1.02)	0.072	22	0.49 (0.26-0.93)	0.029	0.55 (0.28-1.09)	0.090
Social activities in late-life	343	0.48 (0.34-0.69)	<0.001	0.43 (0.29-0.63)	<0.001	29	0.29 (0.15-0.57)	<0.001	0.21 (0.10-0.45)	<0.001
Intellectual activities in midlife	253	0.61 (0.46-0.81)	0.001	0.60 (0.45-0.81)	0.001	23	0.54 (0.29-1.0)	0.053	0.56 (0.29-1.10)	0.098
Intellectual activities in late-life	276	0.55 (0.41-0.74)	<0.001	0.57 (0.42-0.77)	<0.001	20	0.30 (0.16-0.56)	<0.001	0.35 (0.18-0.69)	0.002
Work more than 9 hours in midlife	274	1.04 (0.79-1.37)	0.776	1.02 (0.76-1.37)	0.875	36	2.77 (1.30-5.90)	0.009	2.96 (1.34-6.57)	0.007
Work more than 9 hours in late-life	37	1.15 (0.69-1.92)	0.590	1.31 (0.76-2.26)	0.319	4	1.27 (0.42-3.80)	0.671	2.94 (0.91-9.53)	0.071
HDS in midlife per 1 point increase	-	0.95 (0.90-0.99)	0.035	0.95 (0.90-1.00)	0.074	-	0.85 (0.76-0.95)	0.003	0.86 (0.77-0.96)	0.012
HDS in late-life per 1 point increase	-	0.92 (0.88-0.97)	0.002	0.93 (0.88-0.98)	0.008	-	0.83 (0.75-0.93)	0.001	0.85 (0.75-0.95)	0.008

N, number of participations; OR, odds ratio; CI, confidence interval; HDS, healthy diet score.

* Adjusted for age, gender, education level, housing type, marital status, living condition, number of comorbidities, and SM-MMSE score

When the prefrail and frail subjects were combined into one group and the lifestyle activities were categorized into three levels (never, irregular or daily participation; Supplementary Table 1), daily midlife participation in social activities was found to be associated with a significantly reduced risk of prefrailty/frailty (compared with nonparticipation, adjusted OR=0.59, 95% CI 0.36-0.94, *p*=0.028). Daily (adjusted OR=0.61, 95% CI 0.43-0.85, *p*=0.004) and irregular (adjusted OR=0.59, 95% CI 0.41-0.85, *p*=0.005) midlife participation in intellectual activities correlated negatively with prefrailty/frailty (compared with nonparticipation).

### Sex differences

We then performed a stratified analysis (Table 4), which revealed that the effects of lifestyle activities differed between men and women. For these analyses, frail and prefrail subjects were again combined into one group. Among the women, the risk of prefrailty/frailty was lower for those who participated in late-life social activities (adjusted OR=0.32, 95% CI 0.20-0.52, *p*<0.001), midlife intellectual activities (adjusted OR=0.54, 95% CI 0.38-0.77, *p*=0.001) and late-life intellectual activities (adjusted OR=0.40, 95% CI 0.27-0.58, *p*<0.001) than for those who did not participate. On the contrary, among the men, participation in late-life social activities (adjusted OR=0.61, 95% CI 0.33-1.14, *p*=0.127), midlife intellectual activities (adjusted OR=0.71, 95% CI 0.40-1.24, *p*=0.238) and late-life intellectual activities (adjusted OR=1.03, 95% CI 0.59-1.80, *p*=0.898) had no correlation with prefrailty/frailty. In our earlier analysis, working long hours at midlife was a risk factor for frailty but not for prefrailty; thus, when we combined the prefrail and frail participants into one group and performed a gender-stratified analysis, working long hours did not correlate with prefrailty/frailty in either men or women.

**Table 4:** Gender-stratified association of lifestyle activities with the presence of non-robust status.

**Life style activities, yes vs no**	**Male**		**Female**	
	Adjusted OR* (95%CI)	***p***	Adjusted OR* (95%CI)	***p***
Social activities in midlife	0.54 (0.30-0.99)	0.047	0.80 (0.57-1.13)	0.210
Social activities in late-life	0.61 (0.33-1.14)	0.127	0.32 (0.20-0.52)	<0.001
Intellectual activities in midlife	0.71 (0.40-1.24)	0.238	0.54 (0.38-0.77)	0.001
Intellectual activities in late-life	1.03 (0.59-1.80)	0.898	0.40 (0.27-0.58)	<0.001
Work more than 9 hours in midlife	1.39 (0.73-2.64)	0.307	1.06 (0.76-1.48)	0.715
Work more than 9 hours in late-life	1.93 (0.72-5.13)	0.187	1.23 (0.64-2.36)	0.520

OR was obtained using logistic analysis, depended variable was defined as robust and non-robust (i.e. prefrailty/frailty).

*Adjusted for age, education level, housing type, marry status, living condition, number of comorbidities, SM-MMSE score.

## DISCUSSION

Using a community-based sample of older adults in Singapore, we assessed the relationships of various midlife and late-life activities (social activities, intellectual activities and long work hours) and a healthy dietary pattern with frailty status. We found that social activities and intellectual activities were associated with significantly reduced risks of prefrailty and frailty, and we identified gender differences in these associations.

Significant sociodemographic correlates of frailty in this study included the participant’s age, education level, housing type (as an indicator of socioeconomic status), marital status and living conditions; these factors have also been associated with frailty in previous studies [24–26]. Interestingly, there was no difference in the prevalence of frailty between men and women, consistent with previous findings from Singapore [24, 25, 27, 28], but contrary to the results of most studies from other countries [29]. It has been suggested that women are more likely to become frail because they tend to have lower lean masses, lower strength, greater propensities for sarcopenia and poorer nutrition than men [29]. In our study, a higher proportion of women than men participated in late-life social activities, and a lower percentage of women than men worked more than nine hours per day in midlife (Supplementary Table 2). Differences in participation in these activities may have reduced the susceptibility of women to frailty in our study.

As for medical comorbidities, one-third of the frail participants in this study had more than three chronic diseases, while 22.2% of them had no comorbidities. These results indicate that frailty is distinct from but overlapping with comorbidities. A number of chronic diseases (stroke, cataracts/glaucoma, kidney failure and asthma) were associated with frailty. The associations of frailty with specific chronic diseases have been inconsistent among previous studies (Supplementary Table 3), possibly because the number of affected subjects was simply calculated without consideration for the severity or duration of the disease. Moreover, differences in the definition of frailty, the adjustment for confounders and the sociodemographic characteristics of the study populations may have contributed to inconsistencies among studies. In our study, diabetes, arthritis, hip fracture and cancer exhibited trends of association with frailty, but the results were not statistically significant, possibly due to the relatively small number of cases. Cognitive impairment was more prevalent among those with prefrailty and frailty, in agreement with previous studies [24, 27]. The relationship between frailty and anxiety has rarely been examined in previous studies. Although a relatively small number of subjects reported anxiety symptoms in the present study, the proportion of participants with anxiety was significantly higher in the prefrail and frail groups than in the robust group. Thus, our results revealed additional physical and emotional stressors associated with a prefrail or frail status.

Sociodemographic variables and the number of comorbidities were included as potential confounders when we investigated the associations of three types of lifestyle activities with prefrailty and frailty. It was reasonable to find inverse relationships between frailty and participation in social and intellectual activities, as these activities have been reported to correlate negatively with multiple health-related conditions that are linked to frailty. Participation in social activities was inversely associated with disability in Japan [30, 31] and Western countries [32]. Social activity participation has also been associated with improved cognition and reduced depression [16, 33]. Intellectual activities have been positively associated with cognitive function and negatively associated with disability [9, 20]. Despite growing evidence on the beneficial effects of social and intellectual activities on psychological, physical and cognitive outcomes, the present study is one of the few to explore the effects of such activities on frailty. The mechanisms underlying these associations have not been fully elucidated, but involvement in social/intellectual activities may provide a sense of value, belonging, attachment, self-esteem and self-worth [34], thus enhancing psychological health and potentially improving late-life outcomes [11]. Social and intellectual activities may also exert physiological benefits such as enhancing the humoral immune response [35], reducing systemic chronic inflammation (a major biological factor underlying aging) [36] and altering the brain structure [37–39].

Working is not only a way to make a living, but also an essential part of life with potential effects on health. We found that working long hours in midlife was associated with an increased risk of frailty in the elderly. This finding could be attributed to multiple factors. Firstly, working long hours may be associated with a sedentary lifestyle and a shorter duration of leisure time. In our study, participants who worked long hours in midlife performed less physical activity and participated in fewer social and intellectual activities than those who did not work long hours, both in midlife and in old age (Supplementary Table 4). Secondly, the proportion of individuals who worked long hours in midlife and continued working long hours when they became older (10%) was greater than the proportion of participants who had normal working hours in midlife and worked long hours when they became older (4.3%, *p*=0.002). This suggested that the participants who endured long working hours from midlife to late life may have had a relatively low socioeconomic status or high economic pressure, and thus needed to work long hours even after a normal retirement age. A low socioeconomic status may increase the tendency of adults working long hours to engage in unhealthy behaviors [40]. Thirdly, the incidence of chronic diseases such as cardiovascular disease, stroke and mental disorders has been reported to be greater among those who work long hours [21, 41], so these diseases may have contributed to frailty. These results provide practical information on public health concerns and highlight the importance to increasing one’s physical activity despite being productive in midlife.

In previous studies, gender was found to impact both lifestyle activity participation and the associations between lifestyles and health-related outcomes [42, 43]. Few studies have examined the effects of gender on the relationships between lifestyle activities and frailty. In our study, midlife social activities correlated inversely with frailty in men (*p*=0.047), but not in women (*p*=0.210). On the other hand, midlife intellectual activities correlated inversely with frailty in women (p<0.001), but not in men (*p*=0.127). Inaccuracies in self-reported data may have contributed to these differences. There was an interval of 15+ years between the midlife events and the time of data collection, so error and recall bias to a certain extent were unavoidable. The small number of male participants also may have limited our statistical power in detecting the latter association, if it truly existed in the population.

Regarding late-life activities, we observed significant beneficial effects of participation in social and intellectual activities in women after adjustment for sociodemographic factors and health conditions. However, these associations of late-life activities were not found in men. The impact of gender on the association between social activity and health has been inconsistent in previous studies. Some studies revealed no gender-specific effects on the association between social participation and health in the elderly [16, 30], but other studies revealed that social participation was more beneficial for women than for men [44]. These discrepancies may be due to heterogeneity in the classification and operationalized measurement of social engagement. Lack of detailed surveys on social activities also may have limited the ability to capture gender-based differences.

We found that midlife and late-life intellectual activities were inversely associated with prefrailty/frailty only in women. One reason for this gender difference may be social characteristics related to gender roles. In Chinese society, Confucianism is a major social value demanding that women be responsible for the household while men serve as breadwinners [45]. Although large numbers of women began to enter the job market after the Second World War, women have had to spend more of their leisure time on less-intelligence-stimulating domestic work, while men have had more freedom in how to spend their time after work. We can speculate that women who could free themselves from housework to participate in intellectual activities had better economic backgrounds or social networks than those who could not, while this trend would not apply to men. Another reason might be that the longitudinal trajectories of change in lifestyle activities differ between the genders [46]. Nimrod and Kleiber pointed out that older men were inclined to remain involved in their former activities, while older women were more likely to start new activities. Participating in new activities later in life can be particularly satisfying and meaningful, potentially leading to favorable late-life outcomes [47]. A 12-year follow-up study in Taiwan also indicated that, among elderly adults, engagement in a variety of activities was more beneficial than participation in any single type of activity [48]. Thus, the inverse association between intellectual activities and prefrailty/frailty only in women may have been due to new activities they performed. Unfortunately, due to the cross-sectional nature of the data and the lack of detailed surveys on specific activities, we could not determine whether longitudinal trajectory changes contributed to the gender-specific effects of intellectual activities. Further work is needed to assess the gender differences in lifestyle activity participation and the effects of dynamic variables on frailty.

Importantly, our results revealed an inverse association between the HDS and frailty. Nutritional factors are key determinants of the onset and progression of frailty [49]. Diets with high protein levels and high antioxidant capacities were associated with a low prevalence of frailty in Japanese women [50, 51]. Conversely, low intakes of certain micronutrients have been associated with an increased risk of frailty [52]. In addition to specific nutrients, dietary patterns may modify the state of frailty. For example, greater adherence to the Mediterranean Diet pattern was associated with a lower risk of frailty in Spain [53] and Germany [54], although this association was not found in Hong Kong [22]. This could be explained by differences between the Chinese diet and the Mediterranean diet, as the consumption of olive oil and wine are lower in Hong Kong than in the Mediterranean [22]. In consideration of this, we included certain components of the Mediterranean diet in our HDS, but did not incorporate data on olive oil, grape wine or whole grain consumption.

Among the dietary components used to define our HDS, fruits, green vegetables and nuts are good sources of micronutrients such as vitamin C, vitamin E, β-carotene, folate, unsaturated fatty acids and polyphenols, which can reduce oxidative stress and inflammation. Marine fish and legumes ensure adequate protein intake, which can improve muscle strength and function [55]. In addition to providing high-quality protein, marine fish also provide beneficial unsaturated fatty acids such as docosahexaenoic acid and eicosapentaenoic acid. Low consumption of meat or meat products can reduce the metabolism of L-carnitine by the intestinal microbiota, thus reducing atherosclerosis [56], the common basis of cardiovascular and cerebrovascular diseases. Meat is also the main source of saturated fat, which has been associated with increased levels of inflammatory markers [57] and thus is not recommended as a major component of a healthy diet. In summary, the inverse association of the midlife and late-life HDS with prefrailty and frailty supports the contribution of the diet to frailty and underscores the importance of assessing dietary intake according to local food consumption characteristics.

The present study has expanded the current literature by revealing the associations of the dietary pattern and various lifestyle activities with frailty. However, there are several limitations to this study. Firstly, the cross-sectional nature of the study did not allow us to determine the causal association of the factors with frailty. On one hand, social/intellectual activities may reduce the risk of frailty, but on the other hand, the robust population may be more willing to participate in social/intellectual activities. A second limitation was the measurement of different types of lifestyle activities. The direction and strength of an association depends on the type of activity [9, 18, 58], and without a detailed checklist, we could not determine which activities exerted greater effects than others. Thirdly, our data were based on self-reported recall of chronic diseases, lifestyle activities and food consumption, raising the issue of recall bias. However, the participants were unaware of the group to which they belonged (robust, prefrail or frail), so there was no differential misclassification, and recall bias may have led to underestimation of the true effect. Further analyses with detailed quantitative and qualitative measurements are required to validate our findings.

The present study demonstrated that active participation in social and intellectual activities and adherence to a healthy dietary pattern focused on green vegetables, fruits, nuts, marine fish and legumes were inversely associated with frailty in elderly Singaporean adults. Working long hours in midlife was associated with an increased risk of frailty in the elderly. The associations seemed to differ according to gender.

## MATERIALS AND METHODS

### Participants

The Diet and Healthy Aging (DaHA) Study was an epidemiologic population-based study carried out from 2011 to 2017 to investigate the relationship of Asian diets with health and health-related phenotypes in aging, such as mild cognitive impairment, frailty, late-life depression, late-life anxiety, etc. The study was approved by the Institutional Review Board of the National University of Singapore. Study participants (aged ≥ 60 years) were recruited from geographically defined residential districts in Jurong, western Singapore via door-to-door visits by research nurses. At a community research center, participants were asked to sign informed consent forms and to complete various questionnaires and functional tests administered by trained research staff. We used data from the first 920 DaHA participants for this study. After subjects with incomplete frailty score data were eliminated (n=25), the remaining 895 participants were included in the final analysis.

### Frailty assessment

There are two principal conceptual models to detect frailty in a population: the more widely used phenotype model [2] and the cumulative deficit model [59]. We assessed frailty based on the phenotype model developed by Fried and colleagues, which includes five criteria: shrinking, weakness, exhaustion, slowness and low activity. Participants fulfilling three or more of these criteria were classified as frail, while those fulfilling one or two of the criteria were classified as prefrail, and those fulfilling none of the criteria were classified as robust.

Our operational definitions for the five criteria were as follows:

1. Shrinking was defined as an unintentional weight loss ≥ 4.5 kilograms (kg) in the previous six months, or a body mass index < 18.5 kg/m^2^.
2. Weakness was assessed by grip strength, which was measured in kg with a hand-held dynamometer. Two trials were performed for each hand, and the maximum grip strength from all attempts was used for analyses. Weakness was established for those in the lowest quintile of handgrip performance after adjustment for sex and body mass index [60].
3. Exhaustion was established based on a response of “no” to the following question from the 15-item Geriatric Depression Scale: “Do you feel full of energy?”.
4. Slowness was assessed with a fast gait speed test, and was measured in seconds over a six-meter course. Participants were asked to wait with both feet one meter behind the starting line, and to start walking as fast as possible without running after a verbal command was given. The total time elapsed between the participant crossing the starting line and the finish line was recorded. Two trials were administered, and the faster trial was used in the analyses. Slowness was established for those in the lowest quintile of performance after adjustment for gender and standing height.
5. Low physical activity was determined by a self-reported response of “never or rarely” or “more than once a month but less than once a week” to the question “How often do you participate in physical activities?”.

### Measurements

Medical comorbidities were determined from each participant’s self-reported history of specific diseases (hypertension, high cholesterol, diabetes mellitus, stroke, cardiac disease, cataracts/glaucoma, kidney failure, asthma, chronic obstructive pulmonary disease, arthritis, osteoporosis, hip fracture, gastrointestinal problems, thyroid problems or cancer) or other chronic diseases. Heart attacks, ischemic heart disease, irregular heartbeats, atrial fibrillation and heart failure were considered cardiac diseases.

Cognitive function was assessed with the SM-MMSE [61], which has been validated for local use in older Singaporean adults [62], with a score ≤ 23 indicating cognitive impairment. Anxiety was measured with the 20-item Geriatric Anxiety Inventory based on a cut-off of 10/11 [63], which has been validated to discriminate between those with and without any anxiety disorder.

Lifestyle activities were measured through an interviewer-administered questionnaire with three key questions: “How often do you participate in social activity?”, “How often do you participate in cognitively demanding/intellectual activity?” and “How often do you work more than nine hours a day?”. “Social activity” referred to face-to-face interpersonal communication and activities with non-family members, while “intellectual activity” referred to activities predominantly requiring cognitive effort, such as reading and writing, word or Sudoku games, puzzles and other brain-stimulating activities [64]. “Long working hours” referred to working hours exceeding standard working hours, which differ from country to country. Much of the literature has recognized standard working hours as around 40 hours per week or eight hours per day. However, it was reported that 52 hours of work per week had the best predictive ability for health outcomes [65]. Therefore, to increase our predictive power, we used nine hours per day – one hour more than the widely accepted standard of eight hours per day – as our definition of long working hours. The frequencies of the three activities in late life (i.e., the time when the survey was administered) and at midlife (i.e., at 45 years old) were recorded based on self-report. Responses were provided on a five-point Likert scale: 1. never or rarely, 2. more than once a month but less than once a week, 3. one to three times a week, 4. four to six times a week, 5. daily. The responses were dichotomized as nonparticipation (never or rarely) and participation (from more than once a month to daily) for analysis. The response of participation was recategorized as irregular (from more than once a month to four to six times a week) or daily participation.

Dietary data were collected with a brief food frequency questionnaire that was designed according to habitual consumption of six major food categories: meat, green vegetables, fruits, nuts, marine fish and legumes. The key question was “How often do you consume each of the following foods?”. The consumption of each food category was coded into one of six frequency levels: never or rarely, more than once a month but less than once a week, one to three times a week, four to six times a week, one to two times a day, and three or more times a day. Based on the traditional Mediterranean diet, we defined an operational HDS [66] in which beneficial dietary components (green vegetables, fruits, nuts, marine fish and legumes) were assigned scores of 0-5 when the participant reported consuming them from zero to three or more times per day, respectively. For the consumption of foods presumed to be less healthy (meat and meat products), reverse scoring was implemented, so higher consumption frequencies obtained lower scores (see Supplementary Table 5). All component scores were summed to obtain a total HDS ranging from 0 to 30, with higher values indicating a healthier dietary pattern.

### Other variables

Other measured variables included age, gender, education level (no education, primary education or secondary/higher education), housing type (one- to three-room housing, four- to five-room housing or high-end housing), marital status (married, widowed/divorced/separated), living condition (alone, with spouse, with children or other), smoking (current or past smoking of more than once per month) and alcohol consumption (current or past drinking of more than once per month).

### Statistical analysis

Continuous variables were reported as the mean ± standard deviation. Categorical variables were presented as the frequency and percentage. As the data distribution was skewed, a nonparametric Kruskal-Wallis test was used to determine the differences among the frail, prefrail and robust groups for continuous variables. Chi-square tests were used to test the differences in categorical variables among the three groups. Multinomial logistic regression models were used to determine the OR and 95% Cl for the risk of frailty or prefrailty based on each risk factor, with adjustment for age, gender, education level, housing type, marital status, number of comorbidities and SM-MMSE score as potential confounders. We also adjusted for comorbidities as binary variables (Supplementary Table 6), the results are essentially the same. Correlations between frailty and covariates (Supplementary Table 7) ranged from 0 to 0.513 and were considered as week to moderate correlations. For gender-stratified analyses and comorbidity-stratified analyses (Supplementary Table 8), due to the small number of participants with frailty, we used binary logistic regression after dichotomizing subjects into the robust and non-robust (i.e., prefrail/frail) groups. A two-sided *p* value < 0.05 was considered statistically significant. All analyses were performed with IBM SPSS 22.0 (IBM Corp., Armonk, NY, USA).

## Supplementary Material

Supplementary Tables
