# Supplementary material for: Associations of lifestyle activities and a heathy diet with frailty in old age: a community-based study in Singapore
Source: Aging (Albany NY). 2020 Jan 2;12(1):288–308. doi: 10.18632/aging.102615 (PMC6977658; doi:10.18632/aging.102615)
Supplement: Supplementary Tables [file aging-12-102615-s001..pdf]

SUPPLEMENTARY TABLES

Supplementary Table 1. Association of lifestyle activities and healthy diet score with the presence of non-robust status.

	N	Crude OR (95%CI)	*p*	Adjusted OR* (95%CI)	*p*
Social activities in midlife					
Never	323	1		1	
Irregular	464	0.73 (0.54-0.97)	0.034	0.78 (0.57-1.05)	0.112
Daily	103	0.64 (0.41-1.00)	0.053	0.59 (0.36-0.94)	0.028
Social activities in late-life					
Never	183	1		1	
Irregular	597	0.48 (0.34-0.69)	<0.001	0.44 (0.30-0.64)	<0.001
daily	114	0.33 (0.20-0.55)	<0.001	0.26 (0.15-0.43)	<0.001
Intellectual activities in midlife					
Never	350	1		1	
Irregular	225	0.65 (0.46-0.91)	0.013	0.59 (0.41-0.85)	0.005
daily	310	0.57 (0.42-0.78)	0.001	0.61 (0.43-0.85)	0.004
Intellectual activities in late-life					
Never	305	1		1	
irregular	259	0.57 (0.40-0.80)	0.001	0.56 (0.39-0.80)	0.001
daily	326	0.48 (0.35-0.67)	<0.001	0.53 (0.38-0.75)	<0.001
Work more than 9 hours in midlife					
Never	350	1		1	
Irregular	257	1.06 (0.76-1.46)	0.721	1.03 (0.73-1.46)	0.835
daily	283	1.18 (0.86-1.62)	0.303	1.16 (0.83-1.63)	0.372
Work more than 9 hours in late-life					
Never	823	1		1	
irregular	45	0.90 (0.49-1.65)	0.749	1.06 (0.55-2.02)	0.857
daily	24	1.92 (0.79-4.69)	0.149	2.37 (0.95-5.90)	0.063
HDS in midlife per 1 point increase	-	0.93 (0.89-0.98)	0.011	0.94 (0.90-0.99)	0.033
HDS in late-life per 1 point increase	-	0.91 (0.86-0.95)	<0.001	0.92 (0.87-0.97)	0.003

OR was obtained using logistic analysis, depended variable was defined as robust and non-robust (i.e. prefrailty and frailty).
N, number of participants; OR, odds ratio; CI, confidence interval; HDS, healthy diet score.
* Adjusted for age, gender, education level, housing type, marital status, living condition, number of comorbidities (15), and SM-MMSE score.

Supplementary Table 2. Gender differences in participation in lifestyle activities and the HDS.

	Male	Female	*p**
Social activities in midlife	177(68.3)	390(61.8)	0.066
Social activities in late-life	193(74.2)	518(81.7)	0.012
Intellectual activities in midlife	160(62)	375(59.8)	0.542
Intellectual activities in late-life	170(65.6)	415(65.8)	0.97
Work more than 9 hours in midlife	198(76.2)	342(54.3)	<0.001
Work more than 9 hours in late-life	22(8.5)	47(7.4)	0.603
HDS in midlife	14.6±3.2	14.8±2.7	0.351**
HDS in late-life	14.9±3.0	15.0±2.6	0.375**

Participation was defined more than once a month.
HDS, healthy diet score.
**P*-value obtained using Chi-square test.
** *P*-value obtained using Kruskal Wallis Test.

Supplementary Table 3. Summary of studies on the prevalence and risk factors of frailty in Singapore.

Investigator	Participation (cohort)	Criteria of frailty (prevalence of frailty, prefrailty)	Factors associated with frailty	Specific chronic diseases associated with frailty	Factors not associated with frailty
Liang Feng	1827 Chinese aged 55 and above (SLAS)	Fried criteria (2.5%, 32.4%)	age, education level, alcohol, smoking, comorbidity, cognitive impairment, depression, ADL, IADL.		sex, living alone.
Tze Pin Ng	1685 participants aged 55 and above (SLAS)	Fried criteria (5.3%, 42.3%)	age, education, housing type, ethnicity, living alone, smoking, comorbidity, polypharmacy, cognitive impairment, depression, self-rated health, obesity, high nutritional risk, low albumin, anemia, total cholesterol, white cell count, ALD, IADL, hospital admission.	diabetes, hypertension, cardiovascular disease, coronary heart disease, heart failure, atrial fibrillation, stroke, cataracts/glaucoma, visual impairment, hearing impairment, asthma/COPD, osteoporosis, gastrointestinal problems, chronic kidney disease.	sex, drinking, thyroid disease, arthritis, cancer, orthostatic hypotension, lymphocyte counts.
Liang Feng	1575 Chinese aged 55 and above (SLAS)	Fried criteria (2%, 32%)	age, education, smoking, alcohol, comorbidity, cognitive impairment, depression, ADL-IADL, hospitalization in the past 1 yea, physical component scale.	diabetes, atrial fibrillation, heart failure, cataract and eye disorders, kidney failure, COPD, hip fracture.	sex, hypertension, high cholesterol, heart attack, asthma, arthritis, APOE epsilon4.
Liang Feng	2375 Chinese aged 55 and above (SLAS)	Fried criteria (2.6%, 33.3%)	age, education, smoking, alcohol, comorbidity, cognitive impairment, depression, ADL-IADL disability, physical component score.		sex, APOE epsilon4 carrier, hospitalization in the past year.
Nigel Teo	2406 participants aged 55 and above (SLAS)	Fried criteria (3.4%, 45.1%)	self-defined social frailty (including 7 components: Living alone, no education, absence of a confidant, infrequent contact, infrequent social activities, financial difficulty, socioeconomic deprivation).		
Kai Wei	5685 participants aged 55 and above (SLAS)	Fried criteria (4.5%, 45.7%)	age, ethnicity, sex, education, housing status, comorbidity, polypharmacy, cognitive impairment,	diabetes, hypertension, cardiac disease, stroke, anemia, chronic kidney disease, kidney failure, hearing loss, visual	

			depression, ADL/IADL, hospitalization, quality of life, malnutrition.	impairment.	
Janhavi Ajit Vaingankar	2101 participants aged 60 years and above (Well-being of the Singapore Elderly study)	Fried criteria (5.7%, 45.1%)	age, ethnicity, education, employment status, socioeconomic status, care need, social networks, cognitive impairment, chronic physical condition, hospitalization.	diabetes, hypertension, heart trouble, stroke, transient ischemic attacks, arthritis, visual problems, persistent cough, respiratory problems, stomach or intestinal problems, faints, paralysis, cancer.	sex, marital status, psychological morbidity in caregivers, body mass index, depression, hearing problems, skin conditions.
Reshma Merchant	1051 participants age 65 years and above (Singapore Population Health Studies cohort)	5-item FRAIL scale (6.2%, 37%)	age, sex, ethnicity, comorbidity, polypharmacy, cognitive impairment, depression, Timed-Up and-Go (TUG) test, grip strength, ADL, IADL, subjective health.	diabetes, hypertension, hyperlipidemia, heart attack, stroke.	number of falls, cancer.
Lixia Ge	721 participants aged 60 and above (longitudinal Population Health Index survey)	Clinical Frailty Scale (prevalence of frailty was 24.5%)	age, marital status, education, employment, money insufficiency, living alone, smoking, depression.		sex, ethnicity.

SLAS, Singapore Longitudinal Ageing Studies; COPD, chronic obstructive pulmonary disease; ADL, activities of daily living; IADL, instrumental activities of daily living.

Supplementary Table 4. Association between engagement of midlife long working hours and other lifestyle activities.

	Midlife long working hour		
	**Non-engagement N (%)**	**Engagement N (%)**	***p***
Midlife physical activity			
Non-engagement	120(34.3)	228(42.3)	0.017
Engagement	230(65.7)	311(57.7)	
Late-life physical activity			
Non-engagement	49(14.0)	110(20.4)	0.015
Engagement	301(86.0)	430(79.6)	
Midlife social activity			
Non-engagement	100(28.6)	222(41.4)	<0.001
Engagement	250(71.4)	314(58.6)	
Late-life social activity			
Non-engagement	55(15.7)	128(23.7)	0.004
Engagement	295(84.3)	411(76.3)	
Midlife Intellectual activity			
Non-engagement	109(31.7)	241(44.9)	<0.001
Engagement	235(68.3)	296(55.1)	
Late-life intellectual activity			
Non-engagement	96(27.7)	208(38.6)	0.001
Engagement	250(72.3)	331(61.4)	
Late-life long working hour			
Non-engagement	334(95.7)	485(90.0)	0.002
Engagement	15(4.3)	54(10.0)	

Supplementary Table 5. Operational definition of healthy diet score; the scoring rules for each of the six components.

Dietary item	Frequency of consumption					
	Never or rarely	More than once per month but less than once per week	1-3 times per week	4-6 times per week	1-2 times per day	>2 times per day
Green vegetables	0	1	2	3	4	5
Fruits	0	1	2	3	4	5
Legumes	0	1	2	3	4	5
Nuts	0	1	2	3	4	5
Marine fish	0	1	2	3	4	5
Meat and meat products	5	4	3	2	1	0

Supplementary Table 6. Association of lifestyle activities and healthy diet score with prefrailty and frailty.

	prefrailty				frailty			
Life style activities, yes vs no	Adjusted OR* (95%CI)	*p*	Adjusted OR** (95%CI)	*p*	Adjusted OR* (95%CI)	*p*	Adjusted OR** (95%CI)	*p*
Social activities in midlife	0.76 (0.56-1.02)	0.072	0.76 (0.56-1.03)	0.086	0.55 (0.28-1.09)	0.090	0.55 (0.27-1.11)	0.096
Social activities in late-life	0.43 (0.29-0.63)	<0.001	0.44 (0.30-0.65)	<0.001	0.21 (0.10-0.46)	<0.001	0.22 (0.10-0.47)	<0.001
Intellectual activities in midlife	0.60 (0.45-0.81)	0.001	0.62 (0.46-0.84)	0.003	0.56 (0.29-1.10)	0.098	0.53 (0.26-1.08)	0.081
Intellectual activities in late-life	0.57 (0.42-0.77)	<0.001	0.59 (0.43-0.81)	0.001	0.35 (0.18-0.69)	0.002	0.38 (0.19-0.77)	0.008
Work more than 9 hours in midlife	1.02 (0.76-1.37)	0.875	0.98 (0.73-1.34)	0.944	2.96 (1.34-6.57)	0.007	3.27 (1.44-7.45)	0.005
Work more than 9 hours in late-life	1.31 (0.76-2.26)	0.319	1.32 (0.76-2.30)	0.321	2.94 (0.91-9.53)	0.071	2.81 (0.85-9.27)	0.089
HDS in midlife per 1 point increase	0.95 (0.90-1.00)	0.074	0.95 (0.90-1.01)	0.111	0.86 (0.77-0.96)	0.012	0.86 (0.76-0.96)	0.013
HDS in late-life per 1 point increase	0.93 (0.88-0.98)	0.008	0.93 (0.88-0.98)	0.010	0.85 (0.75-0.95)	0.008	0.83 (0.74-0.94)	0.004

OR, odds ratio; CI, confidence interval; HDS, healthy diet score.

*Adjusted for age, gender, education level, housing type, marital status, living condition, SM-MMSE score, and number of comorbidities,

**Adjusted for age, gender, education level, housing type, marital status, living condition, SM-MMSE score, and comorbidities (including 15 diseases, using binary indicators of each disease).

Supplementary Table 7. Correlation matrix of variables.

	Frailty	Education level	Housing type	Marital status	Living condition	Comorbidities	SM-MMSE
Frailty	1						
Education level	-0.142	1					
Housing type	-0.134	0.135	1				
Marital status	-0.082	0.150	0.219	1			
Living condition	0.037	-0.120	0.098	-0.513	1		
Comorbidities	0.083	-0.057	-0.042	-0.043	0.000	1	
SM-MMSE score	-0.083	0.482	0.078	0.099	-0.129	-0.169*	1

Comorbidities means the number of 15 specific comorbidities.
SM-MMSE means score of SM-MMSE.
Shown are spearman correlation coefficients unless stated otherwise.
* Pearson correlation coefficient.

Supplementary Table 8. Comorbidity-stratified association of lifestyle activities and healthy-diet pattern score with the presence of non-robust status.

**Life style activities, yes vs no**	**No comorbidity**		**Comorbidity**	
	**Adjusted OR* (95%CI)**	***p***	**Adjusted OR* (95%CI)**	***p***
Social activities in midlife	1.16 (0.71-1.89)	0.545	0.55 (0.38-0.81)	0.002
Social activities in late-life	0.38(0.21-0.68)	0.001	0.40 (0.24-0.66)	<0.001
Intellectual activities in midlife	0.83 (0.51-1.34)	0.449	0.49 (0.34-0.72)	<0.001
Intellectual activities in late-life	0.50 (0.30-0.82)	0.007	0.58 (0.40-0.85)	0.006
Work more than 9 hours in midlife	0.97 (0.59-1.59)	0.918	1.12 (0.77-1.62)	0.532
Work more than 9 hours in late-life	1.03 (0.50-2.08)	0.933	2.13 (0.88-5.13)	0.092

OR was obtained using logistic analysis, depended variable was defined as robust and non-robust (i.e. prefrailty/frailty).
No comorbidity means the sum of comorbidity is less than 2.
Comorbidity means the sum of comorbidity is equal or more than 2.
* Adjusted for age, sex, education level, housing type, marry status, living condition, SM-MMSE score.
